# Ethyl 4′-ethenyl-2′-oxo-4-phenyl-2-(3,4,5-trimethoxy­phen­yl)spiro­[pyrrolidine-3,3′-indoline]-5-carboxyl­ate monohydrate

**DOI:** 10.1107/S1600536808031206

**Published:** 2008-10-04

**Authors:** M. Sathyanarayanan, P. Ramesh, Ramalingam Murugan, S. Sriman Narayanan, M. N. Ponnuswamy

**Affiliations:** a PG & Research Department of Physics, A. M. Jain College, Meenambakkam, Chennai 600 114, India; b Department of Physics, Presidency College (Autonomous), Chennai 600 005, India; c Department of Analytical Chemistry, University of Madras, Guindy Campus, Chennai 600 025, India; d Centre of Advanced Study in Crystallography and Biophysics, University of Madras, Guindy Campus, Chennai 600 025, India

## Abstract

In the title compound, C_31_H_32_N_2_O_6_·H_2_O, the pyrrolidine ring adopts an envelope conformation. The ethyl C atoms of the ethoxy­cabonyl group are disordered over two positions with occupancies of *ca* 0.80 and 0.20. Intra­molecular N—H⋯O hydrogen bonds form *S*(5) and *S*(6) ring motifs. Mol­ecules are linked into a three-dimensional framework by O—H⋯O, N—H⋯O and C—H⋯O hydrogen bonds, and by C—H⋯π inter­actions.

## Related literature

For related literature, see: Amalraj *et al.* (2003 ▶); Beddoes *et al.* (1986 ▶); Cordell (1981 ▶); Suzuki *et al.* (1994 ▶). For hydrogen-bond motifs, see: Bernstein *et al.* (1995 ▶). For ring conformational analysis, see: Cremer & Pople (1975 ▶); Nardelli (1983 ▶).
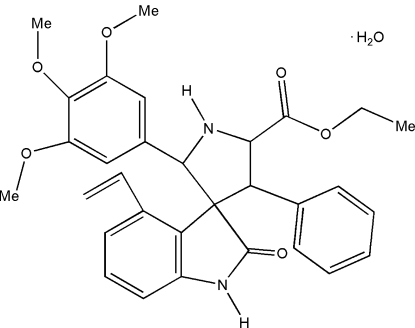


## Experimental

### 

#### Crystal data

C_31_H_32_N_2_O_6_·H_2_O
*M* *_r_* = 546.60
Hexagonal, 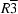

*a* = 38.8029 (10) Å
*c* = 11.0307 (3) Å
*V* = 14383.4 (7) Å^3^
*Z* = 18
Mo *K*α radiation
μ = 0.08 mm^−1^
*T* = 293 (2) K
0.23 × 0.21 × 0.17 mm

#### Data collection

Bruker Kappa APEXII area-detector diffractometer
Absorption correction: multi-scan (*SADABS*, Sheldrick, 2001 ▶) *T*_min_ = 0.982, *T*_max_ = 0.986
111058 measured reflections
7909 independent reflections
5572 reflections with *I* > 2σ(*I*)
*R*_int_ = 0.035

#### Refinement

*R*[*F* ^2^ > 2σ(*F* ^2^)] = 0.046
*wR*(*F* ^2^) = 0.141
*S* = 1.05
7909 reflections
399 parameters
29 restraints
H atoms treated by a mixture of independent and constrained refinement
Δρ_max_ = 0.26 e Å^−3^
Δρ_min_ = −0.21 e Å^−3^

### 

Data collection: *APEX2* (Bruker, 2004 ▶); cell refinement: *APEX2*; data reduction: *SAINT* (Bruker, 2004 ▶); program(s) used to solve structure: *SHELXS97* (Sheldrick, 2008 ▶); program(s) used to refine structure: *SHELXL97* (Sheldrick, 2008 ▶); molecular graphics: *ORTEP-3* (Farrugia, 1997 ▶); software used to prepare material for publication: *SHELXL97* and *PLATON* (Spek, 2003 ▶).

## Supplementary Material

Crystal structure: contains datablocks global, I. DOI: 10.1107/S1600536808031206/ci2651sup1.cif

Structure factors: contains datablocks I. DOI: 10.1107/S1600536808031206/ci2651Isup2.hkl

Additional supplementary materials:  crystallographic information; 3D view; checkCIF report

## Figures and Tables

**Table 1:** Hydrogen-bond geometry (Å, °)

*D*—H⋯*A*	*D*—H	H⋯*A*	*D*⋯*A*	*D*—H⋯*A*
N1—H1⋯O1	0.86 (2)	2.419 (18)	2.8088 (18)	108 (1)
N1—H1⋯O3	0.86 (2)	2.376 (18)	2.9395 (17)	123 (1)
O4—H4*B*⋯O1^i^	0.85 (3)	2.142 (18)	2.909 (2)	150 (3)
N16—H16⋯O3^ii^	0.87 (2)	1.99 (2)	2.8449 (17)	166 (2)
C5—H5⋯O5^iii^	0.98	2.45	3.3197 (18)	147
C18—H18⋯O4^iv^	0.93	2.46	3.357 (2)	162
C24—H24*B*⋯*Cg*1^iii^	0.93	2.93	3.776 (2)	153

Symmetry codes: (i) 
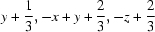
; (ii) 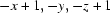; (iii) 
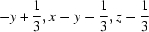
; (iv) 
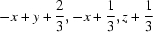
.

**Fig. 1.**
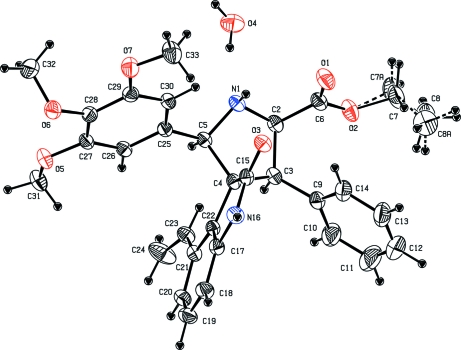
The molecular structure of the title compound, showing 30% probability displacement ellipsoids. Both disorder components are shown.

## supplementary crystallographic information

### Comment

Substituted pyrrolidine compounds possess antimicrobial and antifungal activity against various pathogens (Amalraj *et al.*, 2003). Several optically active pyrrolidine compounds are used as intermediates in controlled asymmetric synthesis (Suzuki *et al.*, 1994). The spiro-indole-pyrrolidine ring system is a frequently encountered structural motif in many biologically important and pharmacologically relevant alkaloids, *e.g.* vincrinstine, vinblastine and spirotypostatins (Cordell, 1981). Against this background and to ascertain the detailed information on its molecular conformation, the structure determination of the title compound has been carried out.

The pyrrolidine ring (N1—C5) adopts an envelope conformation, with puckering (Cremer & Pople, 1975) and asymmetry (Nardelli, 1983) parameters q_2_ = 0.416 (2) Å, φ = 137.5 (2)° and Δ_s_(C5) = 2.8 (2)°. The indoline ring system is planar and the keto atom O3 lies on the plane. The sum of angles at atom N1 of the pyrrolidine ring (323.3°) is in accordance with *sp*^3^ hybridization (Beddoes *et al.*, 1986). The ethoxycarbonyl group is in an extended conformation as evidenced by torsion angles C2—C6—O2—C7 of -170.3 (3)° and C6—O2—C7—C8 of 170.3 (3)°.

Intramolecular N1—H1···O1 and N1—H1···O3 hydrogen bonds generate S(5) and S(6) ring motifs (Bernstein *et al.* 1995), respectively. The crystal packing is stabilized by O—H···O, O—H···N, N—H···O and C—H···O hydrogen bonds, and C—H···π intermolecular interactions (Table 1) which link the molecules into a three-dimensional framework.

### Experimental

3-Arylidene-4-vinylindoline-2-one (0.5 g, 1.0 mol) and (*E*)-ethyl-2-(3,4,5-trimethoxybenzylideneamino)acetate (0.15 g, 1.0 mol) in acetonitrile (10 ml) was stirred in the presence of catalytic amount of AgOAc and triethylamine. The obtained crude product was recrystallized in n-hexane-acetone (8:2 *v*/*v*).

### Refinement

The ethyl C atoms of the ethoxycarbonyl group are disordered over two positions (C7/C7A and C8/C8A) with refined occupancies of 0.797 (8) and 0.203 (8). The corresponding bond distances involving the disordered atoms were restrained to 1.54 (5) Å, and also the U^ij^ parameters of atoms C7, C7A, C8 and C8A were restrained to an approximate isotropic behaviour. The O– and N-bound H atoms were located in a difference map and refined with O—H and H···H distances restrained to 0.84 (1) and 1.37 (1) Å, respectively. The remaining H atoms were positioned geometrically (C—H = 0.93–0.98 Å) and allowed to ride on their parent atoms, with *U*_iso_(H) = 1.2–1.5(methyl) *U*_eq_(C). A search for solvent-accessible voids in the crystal structure using *PLATON* showed a potential solvent volume of 2189.3 Å^3^ and subsequent application of SQUEEZE procedures showed three relevant voids each with a solvent-accessible volume of 730 Å^3^. However, this procedure showed no electrons in the voids. This indicates that the crystal lost nearly all of its solvent of crystallization by the time it was used for data collection, without collapse of the structure.

### Crystal data

**Table:**

C_31_H_32_N_2_O_6_·H_2_O	*D*_x_ = 1.136 Mg m^−^^3^
*M**_r_* = 546.60	Mo *K*α radiation, λ = 0.71073 Å
Hexagonal, *R*3	Cell parameters from 5683 reflections
Hall symbol: -R 3	θ = 1.1–28.2°
*a* = 38.8029 (10) Å	µ = 0.08 mm^−^^1^
*c* = 11.0307 (3) Å	*T* = 293 K
*V* = 14383.4 (7) Å^3^	Block, colourless
*Z* = 18	0.23 × 0.21 × 0.17 mm
*F*(000) = 5220	

### Data collection

**Table:**

Bruker Kappa APEXII area-detector diffractometer	7909 independent reflections
Radiation source: fine-focus sealed tube	5572 reflections with *I* > 2σ(*I*)
graphite	*R*_int_ = 0.035
ω and φ scans	θ_max_ = 28.2°, θ_min_ = 1.9°
Absorption correction: multi-scan (SADABS, Sheldrick, 2001)	*h* = −51→51
*T*_min_ = 0.982, *T*_max_ = 0.986	*k* = −51→51
111058 measured reflections	*l* = −14→14

### Refinement

**Table:**

Refinement on *F*^2^	Primary atom site location: structure-invariant direct methods
Least-squares matrix: full	Secondary atom site location: difference Fourier map
*R*[*F*^2^ > 2σ(*F*^2^)] = 0.046	Hydrogen site location: inferred from neighbouring sites
*wR*(*F*^2^) = 0.142	H atoms treated by a mixture of independent and constrained refinement
*S* = 1.05	*w* = 1/[σ^2^(*F*_o_^2^) + (0.0548*P*)^2^ + 16.9913*P*] where *P* = (*F*_o_^2^ + 2*F*_c_^2^)/3
7909 reflections	(Δ/σ)_max_ = 0.001
399 parameters	Δρ_max_ = 0.26 e Å^−^^3^
29 restraints	Δρ_min_ = −0.21 e Å^−^^3^

### Special details

**Table:**

Geometry. All e.s.d.'s (except the e.s.d. in the dihedral angle between two l.s. planes) are estimated using the full covariance matrix. The cell e.s.d.'s are taken into account individually in the estimation of e.s.d.'s in distances, angles and torsion angles; correlations between e.s.d.'s in cell parameters are only used when they are defined by crystal symmetry. An approximate (isotropic) treatment of cell e.s.d.'s is used for estimating e.s.d.'s involving l.s. planes.
Refinement. Refinement of *F*^2^ against ALL reflections. The weighted *R*-factor *wR* and goodness of fit *S* are based on *F*^2^, conventional *R*-factors *R* are based on *F*, with *F* set to zero for negative *F*^2^. The threshold expression of *F*^2^ > σ(*F*^2^) is used only for calculating *R*-factors(gt) *etc*. and is not relevant to the choice of reflections for refinement. *R*-factors based on *F*^2^ are statistically about twice as large as those based on *F*, and *R*- factors based on ALL data will be even larger.

### Fractional atomic coordinates and isotropic or equivalent isotropic displacement parameters (Å^2^)

**Table:**

	*x*	*y*	*z*	*U*_iso_*/*U*_eq_	Occ. (<1)
O1	0.56007 (4)	0.06180 (4)	0.10291 (14)	0.0633 (4)	
O2	0.56908 (4)	0.12184 (4)	0.05914 (13)	0.0619 (4)	
O3	0.50835 (3)	0.01984 (3)	0.34955 (10)	0.0412 (3)	
O5	0.31168 (3)	−0.07849 (4)	0.31585 (11)	0.0499 (3)	
O6	0.33393 (4)	−0.13259 (3)	0.34016 (11)	0.0501 (3)	
O7	0.40791 (4)	−0.11581 (4)	0.28214 (13)	0.0556 (3)	
N1	0.47767 (4)	0.03182 (4)	0.11926 (11)	0.0385 (3)	
H1	0.4898 (5)	0.0196 (5)	0.1441 (16)	0.041 (5)*	
N16	0.47798 (4)	0.03293 (4)	0.50612 (12)	0.0400 (3)	
H16	0.4835 (5)	0.0200 (6)	0.5595 (18)	0.048 (5)*	
C2	0.50558 (5)	0.07457 (5)	0.12126 (13)	0.0392 (3)	
H2	0.4975	0.0866	0.0572	0.047*	
C3	0.50071 (4)	0.09151 (5)	0.24589 (13)	0.0376 (3)	
H3	0.4867	0.1059	0.2262	0.045*	
C4	0.46974 (4)	0.05359 (4)	0.31510 (12)	0.0326 (3)	
C5	0.44655 (4)	0.02552 (4)	0.20623 (12)	0.0337 (3)	
H5	0.4309	0.0362	0.1690	0.040*	
C6	0.54749 (5)	0.08427 (6)	0.09354 (15)	0.0467 (4)	
C7	0.61222 (16)	0.13632 (13)	0.0495 (5)	0.0722 (13)	0.797 (8)
H7A	0.6178	0.1242	−0.0189	0.087*	0.797 (8)
H7B	0.6221	0.1305	0.1229	0.087*	0.797 (8)
C8	0.63069 (12)	0.18077 (11)	0.0316 (6)	0.121 (2)	0.797 (8)
H8A	0.6259	0.1860	−0.0499	0.182*	0.797 (8)
H8B	0.6588	0.1935	0.0456	0.182*	0.797 (8)
H8C	0.6191	0.1910	0.0876	0.182*	0.797 (8)
C7A	0.6071 (7)	0.1388 (7)	0.0101 (16)	0.068 (5)	0.203 (8)
H7C	0.6102	0.1568	−0.0554	0.081*	0.203 (8)
H7D	0.6121	0.1184	−0.0212	0.081*	0.203 (8)
C8A	0.6359 (5)	0.1615 (6)	0.1138 (16)	0.109 (6)	0.203 (8)
H8D	0.6324	0.1434	0.1780	0.163*	0.203 (8)
H8E	0.6305	0.1816	0.1439	0.163*	0.203 (8)
H8F	0.6628	0.1739	0.0846	0.163*	0.203 (8)
C9	0.53732 (5)	0.12089 (5)	0.31560 (16)	0.0463 (4)	
C10	0.54234 (7)	0.15785 (7)	0.3395 (3)	0.0837 (8)	
H10	0.5233	0.1640	0.3128	0.100*	
C11	0.57526 (10)	0.18618 (9)	0.4028 (4)	0.1240 (13)	
H11	0.5782	0.2111	0.4177	0.149*	
C12	0.60336 (9)	0.17751 (10)	0.4432 (3)	0.1093 (11)	
H12	0.6255	0.1965	0.4855	0.131*	
C13	0.59885 (7)	0.14127 (8)	0.4215 (2)	0.0780 (7)	
H13	0.6178	0.1353	0.4496	0.094*	
C14	0.56632 (5)	0.11301 (6)	0.35802 (17)	0.0554 (5)	
H14	0.5638	0.0882	0.3434	0.066*	
C15	0.48836 (4)	0.03372 (4)	0.38989 (13)	0.0345 (3)	
C17	0.45453 (4)	0.05069 (5)	0.52273 (13)	0.0370 (3)	
C18	0.44120 (5)	0.05730 (5)	0.63084 (14)	0.0469 (4)	
H18	0.4468	0.0495	0.7044	0.056*	
C19	0.41904 (5)	0.07617 (6)	0.62473 (15)	0.0507 (4)	
H19	0.4099	0.0816	0.6960	0.061*	
C20	0.41024 (5)	0.08710 (5)	0.51543 (15)	0.0446 (4)	
H20	0.3951	0.0996	0.5148	0.054*	
C21	0.42347 (4)	0.07994 (4)	0.40474 (13)	0.0366 (3)	
C22	0.44694 (4)	0.06215 (4)	0.41045 (12)	0.0329 (3)	
C23	0.41162 (5)	0.09042 (5)	0.28964 (15)	0.0430 (4)	
H23	0.4249	0.0898	0.2202	0.052*	
C24	0.38483 (8)	0.10039 (9)	0.2753 (2)	0.0826 (8)	
H24A	0.3707	0.1015	0.3419	0.099*	
H24B	0.3796	0.1065	0.1984	0.099*	
C25	0.41775 (4)	−0.01738 (4)	0.23731 (12)	0.0344 (3)	
C26	0.37872 (4)	−0.02733 (5)	0.26238 (13)	0.0363 (3)	
H26	0.3714	−0.0079	0.2588	0.044*	
C27	0.35070 (4)	−0.06595 (5)	0.29254 (13)	0.0377 (3)	
C28	0.36146 (5)	−0.09500 (5)	0.30097 (14)	0.0392 (3)	
C29	0.40046 (5)	−0.08511 (5)	0.27477 (14)	0.0401 (3)	
C30	0.42853 (5)	−0.04645 (5)	0.24184 (14)	0.0396 (3)	
H30	0.4544	−0.0401	0.2230	0.048*	
C31	0.29816 (5)	−0.05142 (6)	0.29070 (19)	0.0546 (5)	
H31A	0.2701	−0.0640	0.3062	0.082*	
H31B	0.3032	−0.0435	0.2072	0.082*	
H31C	0.3120	−0.0284	0.3416	0.082*	
C32	0.31732 (7)	−0.16139 (7)	0.2477 (2)	0.0876 (9)	
H32A	0.2983	−0.1865	0.2822	0.131*	
H32B	0.3380	−0.1639	0.2085	0.131*	
H32C	0.3044	−0.1533	0.1894	0.131*	
C33	0.44799 (6)	−0.10687 (6)	0.2791 (2)	0.0670 (6)	
H33A	0.4490	−0.1309	0.2889	0.100*	
H33B	0.4625	−0.0888	0.3437	0.100*	
H33C	0.4597	−0.0948	0.2028	0.100*	
O4	0.31014 (5)	0.11716 (5)	0.52433 (13)	0.0667 (4)	
H4A	0.3088 (8)	0.1132 (9)	0.6004 (10)	0.110 (11)*	
H4B	0.3348 (4)	0.1306 (9)	0.507 (2)	0.122 (12)*	

### Atomic displacement parameters (Å^2^)

**Table:**

	*U*^11^	*U*^22^	*U*^33^	*U*^12^	*U*^13^	*U*^23^
O1	0.0469 (7)	0.0699 (9)	0.0813 (10)	0.0354 (7)	0.0131 (7)	0.0068 (7)
O2	0.0454 (7)	0.0612 (8)	0.0703 (9)	0.0201 (6)	0.0217 (6)	0.0187 (7)
O3	0.0417 (6)	0.0510 (7)	0.0404 (6)	0.0303 (5)	0.0012 (5)	0.0064 (5)
O5	0.0351 (6)	0.0546 (7)	0.0608 (7)	0.0231 (6)	0.0113 (5)	0.0135 (6)
O6	0.0465 (7)	0.0408 (6)	0.0553 (7)	0.0161 (5)	0.0091 (5)	0.0048 (5)
O7	0.0459 (7)	0.0424 (7)	0.0841 (9)	0.0262 (6)	0.0031 (6)	0.0014 (6)
N1	0.0389 (7)	0.0463 (8)	0.0349 (6)	0.0247 (6)	0.0067 (5)	0.0077 (5)
N16	0.0442 (7)	0.0492 (8)	0.0330 (6)	0.0281 (7)	−0.0007 (5)	0.0077 (6)
C2	0.0381 (8)	0.0459 (9)	0.0357 (7)	0.0226 (7)	0.0052 (6)	0.0112 (6)
C3	0.0355 (8)	0.0409 (8)	0.0398 (8)	0.0218 (7)	0.0056 (6)	0.0095 (6)
C4	0.0295 (7)	0.0374 (8)	0.0331 (7)	0.0184 (6)	0.0013 (5)	0.0054 (6)
C5	0.0321 (7)	0.0427 (8)	0.0307 (7)	0.0219 (7)	0.0010 (5)	0.0049 (6)
C6	0.0409 (9)	0.0566 (11)	0.0407 (8)	0.0231 (8)	0.0088 (7)	0.0068 (7)
C7	0.043 (2)	0.079 (2)	0.078 (3)	0.0183 (18)	0.019 (2)	0.013 (2)
C8	0.073 (2)	0.080 (3)	0.164 (5)	0.0039 (19)	0.033 (3)	0.003 (3)
C7A	0.050 (7)	0.077 (8)	0.069 (8)	0.026 (5)	0.003 (6)	0.024 (6)
C8A	0.089 (8)	0.114 (10)	0.111 (10)	0.042 (7)	0.006 (7)	−0.015 (7)
C9	0.0394 (9)	0.0424 (9)	0.0493 (9)	0.0146 (7)	0.0091 (7)	0.0039 (7)
C10	0.0583 (13)	0.0541 (13)	0.134 (2)	0.0244 (11)	−0.0018 (14)	−0.0212 (14)
C11	0.085 (2)	0.0677 (18)	0.199 (4)	0.0227 (16)	−0.010 (2)	−0.057 (2)
C12	0.0620 (16)	0.101 (2)	0.129 (3)	0.0137 (16)	−0.0146 (16)	−0.050 (2)
C13	0.0464 (12)	0.0876 (18)	0.0723 (14)	0.0127 (11)	−0.0080 (10)	−0.0029 (12)
C14	0.0428 (10)	0.0570 (11)	0.0559 (10)	0.0170 (9)	−0.0032 (8)	0.0038 (8)
C15	0.0306 (7)	0.0368 (8)	0.0356 (7)	0.0165 (6)	−0.0016 (5)	0.0049 (6)
C17	0.0352 (8)	0.0393 (8)	0.0357 (7)	0.0181 (7)	0.0010 (6)	0.0039 (6)
C18	0.0512 (10)	0.0565 (10)	0.0331 (7)	0.0271 (8)	0.0021 (7)	0.0032 (7)
C19	0.0534 (10)	0.0622 (11)	0.0385 (8)	0.0304 (9)	0.0083 (7)	−0.0018 (7)
C20	0.0424 (9)	0.0494 (9)	0.0472 (9)	0.0268 (8)	0.0073 (7)	0.0005 (7)
C21	0.0333 (7)	0.0380 (8)	0.0387 (7)	0.0180 (6)	0.0038 (6)	0.0037 (6)
C22	0.0305 (7)	0.0347 (7)	0.0322 (7)	0.0154 (6)	0.0020 (5)	0.0036 (5)
C23	0.0446 (9)	0.0526 (10)	0.0425 (8)	0.0324 (8)	0.0042 (7)	0.0052 (7)
C24	0.0934 (17)	0.149 (2)	0.0544 (12)	0.0973 (19)	0.0075 (11)	0.0120 (13)
C25	0.0329 (7)	0.0418 (8)	0.0292 (6)	0.0193 (6)	−0.0002 (5)	0.0013 (6)
C26	0.0367 (8)	0.0432 (8)	0.0340 (7)	0.0236 (7)	0.0018 (6)	0.0034 (6)
C27	0.0317 (7)	0.0483 (9)	0.0321 (7)	0.0194 (7)	0.0031 (5)	0.0020 (6)
C28	0.0381 (8)	0.0400 (8)	0.0355 (7)	0.0164 (7)	0.0023 (6)	0.0005 (6)
C29	0.0406 (8)	0.0410 (8)	0.0418 (8)	0.0226 (7)	−0.0008 (6)	−0.0028 (6)
C30	0.0334 (8)	0.0447 (9)	0.0432 (8)	0.0213 (7)	0.0017 (6)	−0.0012 (6)
C31	0.0411 (9)	0.0628 (12)	0.0665 (11)	0.0310 (9)	0.0115 (8)	0.0136 (9)
C32	0.0689 (15)	0.0558 (13)	0.0984 (18)	0.0013 (11)	0.0245 (13)	−0.0236 (12)
C33	0.0528 (12)	0.0571 (12)	0.1029 (17)	0.0365 (10)	0.0078 (11)	0.0063 (11)
O4	0.0785 (11)	0.0625 (9)	0.0496 (8)	0.0280 (8)	0.0013 (7)	0.0025 (7)

### Geometric parameters (Å, °)

**Table:**

O1—C6	1.198 (2)	C10—C11	1.387 (4)
O2—C6	1.323 (2)	C10—H10	0.93
O2—C7A	1.39 (2)	C11—C12	1.367 (5)
O2—C7	1.479 (6)	C11—H11	0.93
O3—C15	1.2281 (18)	C12—C13	1.349 (4)
O5—C27	1.3632 (18)	C12—H12	0.93
O5—C31	1.417 (2)	C13—C14	1.379 (3)
O6—C28	1.3774 (19)	C13—H13	0.93
O6—C32	1.409 (3)	C14—H14	0.93
O7—C29	1.3618 (19)	C17—C18	1.374 (2)
O7—C33	1.414 (2)	C17—C22	1.396 (2)
N1—C2	1.459 (2)	C18—C19	1.382 (3)
N1—C5	1.4639 (18)	C18—H18	0.93
N1—H1	0.864 (19)	C19—C20	1.377 (2)
N16—C15	1.3397 (19)	C19—H19	0.93
N16—C17	1.401 (2)	C20—C21	1.405 (2)
N16—H16	0.87 (2)	C20—H20	0.93
C2—C6	1.506 (2)	C21—C22	1.392 (2)
C2—C3	1.575 (2)	C21—C23	1.475 (2)
C2—H2	0.98	C23—C24	1.288 (3)
C3—C9	1.513 (2)	C23—H23	0.93
C3—C4	1.557 (2)	C24—H24A	0.93
C3—H3	0.98	C24—H24B	0.93
C4—C22	1.514 (2)	C25—C30	1.386 (2)
C4—C15	1.5344 (19)	C25—C26	1.391 (2)
C4—C5	1.568 (2)	C26—C27	1.382 (2)
C5—C25	1.509 (2)	C26—H26	0.93
C5—H5	0.98	C27—C28	1.387 (2)
C7—C8	1.514 (4)	C28—C29	1.393 (2)
C7—H7A	0.97	C29—C30	1.391 (2)
C7—H7B	0.97	C30—H30	0.93
C8—H8A	0.96	C31—H31A	0.96
C8—H8B	0.96	C31—H31B	0.96
C8—H8C	0.96	C31—H31C	0.96
C7A—C8A	1.532 (5)	C32—H32A	0.96
C7A—H7C	0.97	C32—H32B	0.96
C7A—H7D	0.97	C32—H32C	0.96
C8A—H8D	0.96	C33—H33A	0.96
C8A—H8E	0.96	C33—H33B	0.96
C8A—H8F	0.96	C33—H33C	0.96
C9—C10	1.373 (3)	O4—H4A	0.850 (10)
C9—C14	1.387 (3)	O4—H4B	0.85 (3)
			
C6—O2—C7A	124.8 (11)	C13—C12—H12	120.2
C6—O2—C7	114.3 (2)	C11—C12—H12	120.2
C7A—O2—C7	20.0 (6)	C12—C13—C14	120.6 (3)
C27—O5—C31	117.10 (13)	C12—C13—H13	119.7
C28—O6—C32	114.69 (15)	C14—C13—H13	119.7
C29—O7—C33	118.19 (14)	C13—C14—C9	121.1 (2)
C2—N1—C5	105.16 (12)	C13—C14—H14	119.4
C2—N1—H1	108.6 (12)	C9—C14—H14	119.4
C5—N1—H1	109.4 (12)	O3—C15—N16	125.83 (13)
C15—N16—C17	112.06 (12)	O3—C15—C4	125.81 (13)
C15—N16—H16	120.3 (13)	N16—C15—C4	108.32 (12)
C17—N16—H16	127.2 (13)	C18—C17—C22	123.46 (15)
N1—C2—C6	112.10 (14)	C18—C17—N16	127.07 (14)
N1—C2—C3	108.17 (11)	C22—C17—N16	109.45 (13)
C6—C2—C3	114.37 (13)	C17—C18—C19	116.65 (15)
N1—C2—H2	107.3	C17—C18—H18	121.7
C6—C2—H2	107.3	C19—C18—H18	121.7
C3—C2—H2	107.3	C20—C19—C18	121.46 (15)
C9—C3—C4	116.95 (12)	C20—C19—H19	119.3
C9—C3—C2	119.65 (13)	C18—C19—H19	119.3
C4—C3—C2	103.33 (12)	C19—C20—C21	121.92 (16)
C9—C3—H3	105.2	C19—C20—H20	119.0
C4—C3—H3	105.2	C21—C20—H20	119.0
C2—C3—H3	105.2	C22—C21—C20	116.93 (14)
C22—C4—C15	102.14 (11)	C22—C21—C23	123.17 (13)
C22—C4—C3	113.14 (12)	C20—C21—C23	119.89 (14)
C15—C4—C3	113.80 (12)	C21—C22—C17	119.51 (13)
C22—C4—C5	119.27 (12)	C21—C22—C4	132.54 (13)
C15—C4—C5	108.28 (12)	C17—C22—C4	107.86 (12)
C3—C4—C5	100.64 (11)	C24—C23—C21	126.75 (17)
N1—C5—C25	115.29 (12)	C24—C23—H23	116.6
N1—C5—C4	104.29 (11)	C21—C23—H23	116.6
C25—C5—C4	116.34 (11)	C23—C24—H24A	120.0
N1—C5—H5	106.8	C23—C24—H24B	120.0
C25—C5—H5	106.8	H24A—C24—H24B	120.0
C4—C5—H5	106.8	C30—C25—C26	119.79 (14)
O1—C6—O2	124.28 (16)	C30—C25—C5	123.19 (13)
O1—C6—C2	125.63 (16)	C26—C25—C5	117.02 (13)
O2—C6—C2	110.08 (15)	C27—C26—C25	120.38 (14)
O2—C7—C8	104.1 (4)	C27—C26—H26	119.8
O2—C7—H7A	110.9	C25—C26—H26	119.8
C8—C7—H7A	110.9	O5—C27—C26	124.30 (14)
O2—C7—H7B	110.9	O5—C27—C28	115.41 (14)
C8—C7—H7B	110.9	C26—C27—C28	120.29 (14)
H7A—C7—H7B	109.0	O6—C28—C27	119.52 (14)
C7—C8—H8A	109.5	O6—C28—C29	121.14 (15)
C7—C8—H8B	109.5	C27—C28—C29	119.28 (14)
H8A—C8—H8B	109.5	O7—C29—C30	124.38 (14)
C7—C8—H8C	109.5	O7—C29—C28	115.04 (14)
H8A—C8—H8C	109.5	C30—C29—C28	120.56 (14)
H8B—C8—H8C	109.5	C25—C30—C29	119.65 (14)
O2—C7A—C8A	106.2 (15)	C25—C30—H30	120.2
O2—C7A—H7C	110.5	C29—C30—H30	120.2
C8A—C7A—H7C	110.5	O5—C31—H31A	109.5
O2—C7A—H7D	110.5	O5—C31—H31B	109.5
C8A—C7A—H7D	110.5	H31A—C31—H31B	109.5
H7C—C7A—H7D	108.7	O5—C31—H31C	109.5
C7A—C8A—H8D	109.5	H31A—C31—H31C	109.5
C7A—C8A—H8E	109.5	H31B—C31—H31C	109.5
H8D—C8A—H8E	109.5	O6—C32—H32A	109.5
C7A—C8A—H8F	109.5	O6—C32—H32B	109.5
H8D—C8A—H8F	109.5	H32A—C32—H32B	109.5
H8E—C8A—H8F	109.5	O6—C32—H32C	109.5
C10—C9—C14	117.30 (19)	H32A—C32—H32C	109.5
C10—C9—C3	118.31 (18)	H32B—C32—H32C	109.5
C14—C9—C3	124.39 (16)	O7—C33—H33A	109.5
C9—C10—C11	121.2 (3)	O7—C33—H33B	109.5
C9—C10—H10	119.4	H33A—C33—H33B	109.5
C11—C10—H10	119.4	O7—C33—H33C	109.5
C12—C11—C10	120.1 (3)	H33A—C33—H33C	109.5
C12—C11—H11	120.0	H33B—C33—H33C	109.5
C10—C11—H11	120.0	H4A—O4—H4B	106.1 (15)
C13—C12—C11	119.7 (3)		
			
C5—N1—C2—C6	149.93 (13)	C5—C4—C15—N16	−128.16 (13)
C5—N1—C2—C3	22.92 (15)	C15—N16—C17—C18	−175.19 (16)
N1—C2—C3—C9	135.98 (14)	C15—N16—C17—C22	3.36 (18)
C6—C2—C3—C9	10.3 (2)	C22—C17—C18—C19	0.3 (3)
N1—C2—C3—C4	3.84 (15)	N16—C17—C18—C19	178.67 (16)
C6—C2—C3—C4	−121.84 (14)	C17—C18—C19—C20	1.1 (3)
C9—C3—C4—C22	71.14 (17)	C18—C19—C20—C21	−0.4 (3)
C2—C3—C4—C22	−155.16 (12)	C19—C20—C21—C22	−1.7 (2)
C9—C3—C4—C15	−44.87 (18)	C19—C20—C21—C23	177.00 (16)
C2—C3—C4—C15	88.82 (14)	C20—C21—C22—C17	3.0 (2)
C9—C3—C4—C5	−160.45 (13)	C23—C21—C22—C17	−175.60 (15)
C2—C3—C4—C5	−26.75 (13)	C20—C21—C22—C4	−172.97 (15)
C2—N1—C5—C25	−169.62 (12)	C23—C21—C22—C4	8.4 (3)
C2—N1—C5—C4	−40.81 (14)	C18—C17—C22—C21	−2.5 (2)
C22—C4—C5—N1	166.34 (12)	N16—C17—C22—C21	178.91 (13)
C15—C4—C5—N1	−77.61 (13)	C18—C17—C22—C4	174.44 (15)
C3—C4—C5—N1	42.03 (13)	N16—C17—C22—C4	−4.17 (17)
C22—C4—C5—C25	−65.48 (17)	C15—C4—C22—C21	179.73 (16)
C15—C4—C5—C25	50.57 (16)	C3—C4—C22—C21	57.0 (2)
C3—C4—C5—C25	170.21 (12)	C5—C4—C22—C21	−61.0 (2)
C7A—O2—C6—O1	11.2 (8)	C15—C4—C22—C17	3.37 (15)
C7—O2—C6—O1	−8.5 (4)	C3—C4—C22—C17	−119.37 (13)
C7A—O2—C6—C2	−170.0 (7)	C5—C4—C22—C17	122.61 (14)
C7—O2—C6—C2	170.3 (3)	C22—C21—C23—C24	167.1 (2)
N1—C2—C6—O1	−20.7 (2)	C20—C21—C23—C24	−11.5 (3)
C3—C2—C6—O1	102.9 (2)	N1—C5—C25—C30	33.13 (19)
N1—C2—C6—O2	160.55 (14)	C4—C5—C25—C30	−89.46 (17)
C3—C2—C6—O2	−75.85 (17)	N1—C5—C25—C26	−146.98 (13)
C6—O2—C7—C8	−170.3 (4)	C4—C5—C25—C26	90.44 (16)
C7A—O2—C7—C8	64 (3)	C30—C25—C26—C27	0.4 (2)
C6—O2—C7A—C8A	−100.1 (19)	C5—C25—C26—C27	−179.48 (13)
C7—O2—C7A—C8A	−36 (2)	C31—O5—C27—C26	9.3 (2)
C4—C3—C9—C10	−113.8 (2)	C31—O5—C27—C28	−170.80 (15)
C2—C3—C9—C10	120.2 (2)	C25—C26—C27—O5	−178.51 (14)
C4—C3—C9—C14	65.8 (2)	C25—C26—C27—C28	1.6 (2)
C2—C3—C9—C14	−60.2 (2)	C32—O6—C28—C27	103.6 (2)
C14—C9—C10—C11	0.5 (4)	C32—O6—C28—C29	−79.3 (2)
C3—C9—C10—C11	−179.9 (3)	O5—C27—C28—O6	−4.9 (2)
C9—C10—C11—C12	−0.4 (5)	C26—C27—C28—O6	175.03 (14)
C10—C11—C12—C13	−0.2 (6)	O5—C27—C28—C29	177.94 (14)
C11—C12—C13—C14	0.6 (5)	C26—C27—C28—C29	−2.1 (2)
C12—C13—C14—C9	−0.4 (4)	C33—O7—C29—C30	12.7 (3)
C10—C9—C14—C13	−0.1 (3)	C33—O7—C29—C28	−168.46 (17)
C3—C9—C14—C13	−179.70 (18)	O6—C28—C29—O7	4.7 (2)
C17—N16—C15—O3	−179.00 (15)	C27—C28—C29—O7	−178.19 (14)
C17—N16—C15—C4	−1.03 (17)	O6—C28—C29—C30	−176.37 (14)
C22—C4—C15—O3	176.54 (14)	C27—C28—C29—C30	0.7 (2)
C3—C4—C15—O3	−61.2 (2)	C26—C25—C30—C29	−1.8 (2)
C5—C4—C15—O3	49.82 (19)	C5—C25—C30—C29	178.09 (14)
C22—C4—C15—N16	−1.44 (15)	O7—C29—C30—C25	−179.95 (15)
C3—C4—C15—N16	120.85 (14)	C28—C29—C30—C25	1.2 (2)

### Hydrogen-bond geometry (Å, °)

**Table:**

*D*—H···*A*	*D*—H	H···*A*	*D*···*A*	*D*—H···*A*
N1—H1···O1	0.86 (2)	2.419 (18)	2.8088 (18)	108 (1)
N1—H1···O3	0.86 (2)	2.376 (18)	2.9395 (17)	123 (1)
O4—H4B···O1^i^	0.85 (3)	2.14 (2)	2.909 (2)	150 (3)
N16—H16···O3^ii^	0.87 (2)	1.99 (2)	2.8449 (17)	166 (2)
C5—H5···O5^iii^	0.98	2.45	3.3197 (18)	147
C18—H18···O4^iv^	0.93	2.46	3.357 (2)	162
C24—H24B···Cg1^iii^	0.93	2.93	3.776 (2)	153

Symmetry codes:  (i) *y*+1/3, −*x*+*y*+2/3, −*z*+2/3; (ii) −*x*+1, −*y*, −*z*+1; (iii) −*y*+1/3, *x*−*y*−1/3, *z*−1/3; (iv) −*x*+*y*+2/3, −*x*+1/3, *z*+1/3.
